# The Migrating Stent: A Case Report of a Patient With Circulation Issues Who Experienced Stent Migration to the Right Ventricle of the Heart

**DOI:** 10.7759/cureus.61575

**Published:** 2024-06-03

**Authors:** Emily Burbank, Kevin Tice

**Affiliations:** 1 Cardiovascular Medicine, Edward Via College of Osteopathic Medicine, Auburn, USA; 2 Internal Medicine, Grandview Medical Center, Birmingham, USA

**Keywords:** chronic venous insufficiency, endovascular stenting, venous doppler, iliofemoral vein thrombosis, iliac stent migration

## Abstract

Over the past several decades, percutaneous venous stenting has surfaced as the forefront for addressing symptomatic venous outflow obstruction. Stent migration is a very rare, but serious life-threatening complication that can occur with the placement of iliofemoral stents. Life-threatening complications following stent migration include but are not limited to damaged valves, arrhythmias, endocarditis, tamponade, and acute heart failure. Stent failure is seldom understood, but one can attribute it to the incorrect stent and or vein sizing and or the inherent natural forces of the body during respiration. Intravascular ultrasound (IVUS) should be utilized for proper vein and stent sizing prior to placement and patients should be monitored more closely after the procedure. Stent retrieval can be very difficult, as the procedure must consider the location of the migrated stent and the comorbidities associated with the patient. This case report explains an 81-year-old Caucasian male who presented to the Emergency Department with dizziness and dyspnea on exertion. Upon further evaluation via transesophageal echocardiogram, he was found to have severe tricuspid regurgitation and an iliofemoral venous stent located in the right ventricle of the heart.

## Introduction

Iliofemoral venous obstruction has been implicated as a causal factor in patients with both post-thrombotic syndrome and non-thrombotic chronic venous insufficiency [1]. Various factors can lead to iliofemoral venous outflow obstruction: malignancy, anatomical variants (May-Thurner syndrome), and acute or chronic deep vein thrombosis (DVT) [1]. Symptoms experienced by patients differ based on the underlying cause. Patients with DVTs typically experience severe swelling, redness, and pain in the lower extremity, whereas patients with post-thrombotic or non-thrombotic syndromes present with more long-term compressive symptoms, such as leg edema, redness, ulceration, and, to some extent, pain [1,2]. Conservative management includes compression stockings, leg elevation, walking, and antithrombotic therapy [3]. Those who fail conservative measures seek other treatment options, such as endovascular iliofemoral stenting. Venous stenting for chronic iliofemoral venous obstruction has become the first-line therapy in those who fail conservative measures and those with quality-of-life impairing clinical manifestations [3].

In numerous studies, stent placement has been proven to be safe and efficacious in patients who have failed conservative therapy with long-term thrombotic and non-thrombotic syndromes [1-7]. According to Jayaraja et al., over the past 20 years, multiple studies and publications have demonstrated the safety and efficacy of stent placement and the long-term outcomes of symptom improvement [3]. Razavi et al. studied the safety and effectiveness of stent placement in patients with iliofemoral venous outflow obstructions. Data were collected over five years (a total of 37 studies reporting 45 treatments) from 2,869 patients. The technical success of stent placement and proper positioning was compared among groups ranging from 94% to 96%. Complication rates were less than 1% and included pulmonary embolism, bleeding, periprocedural mortality, and early thrombosis [1]. There were no reports of stent migration. In a study performed by Knuttinen et al., 70 patients with endovascular occlusion underwent 77 lower extremity interventions. The overall primary patency over 36 months ranged from 91% to 95% with no reports of stent migration. In this study's conclusion, endovascular stenting proved to be safe and durable for up to 36 months in the post-thrombotic patient [4]. Another study performed by Bikenr et al. investigated the patency rate of stent placement in eight patients. A primary patency rate of 100% was observed during an average follow-up of three years (range: 10-121 months) with no procedural or stent-related complications reported [5].

As with any procedure, complications may arise at any point during and after the procedure. One rare but extremely serious, life-threatening complication of stent placement is stent migration. Stent migration is a dislodgment of the endoprosthesis from the primary intended site of deployment [6]. In a systematic review of endovascular stenting in patients with chronic venous disease secondary to obstruction, stent migration occurred in four studies, ranging from 0.9% to 4.3%, with the highest incidence of stent migration to the iliocaval segments, followed by the central and renal veins [7]. Another case report and literature review by Mando et al. found six manuscripts on PubMed and Medline (4/6 describing stent migration) that described the varying frequencies of stent migration ranging from 1.4% to 6.25%. Three patients out of eleven identified as having stent migration to the heart with serious, life-threatening complications requiring open-heart surgery [8].

Complications of stent migration include, but are not limited to, pulmonary embolism, endocarditis, arrhythmias, tamponade, acute heart failure, severe regurgitation, and certain valvopathies [9]. Patients with such complications are at high risk for fatality. In this case report, we present a patient with a large-sized migrated iliofemoral venous stent (16x18) into the right ventricle of the heart. This finding was found when the patient presented to the Emergency Department (ED) with dizziness and shortness of breath in addition to severe tricuspid regurgitation.

## Case presentation

Our patient is an 81-year-old Caucasian male who presented to the ED complaining of dizziness and shortness of breath. The patient endorsed progressively worsening dyspnea on exertion and exercise intolerance. He was seen by his cardiologist the day prior to his ED visit complaining of similar symptoms. His cardiologist performed a transthoracic echocardiogram (TTE), which showed severe tricuspid regurgitation and an echoic imaging spot underneath the tricuspid valve within the right ventricle. The patient did not have the actual imaging and video of the TTE; he just had the written report.

Due to logistical and scheduling difficulties, the patient was unable to have a TEE in the office. The patient was told by the cardiologist to go to the ED immediately due to concerns about iliac stent migration.

In addition to his dizziness and shortness of breath, the patient was also experiencing increased fluid retention. The patient states that he had gained six pounds in two weeks. He stated that, over the past couple of weeks, he has noticed that his pants getting tighter. He has also experienced generalized weakness, chest pain, and lightheadedness.

Past medical history included congestive heart failure, chronic obstructive pulmonary disease, chronic kidney disease stage 3b (CKD-3b), benign prostatic hyperplasia, type II diabetes mellitus, peripheral artery disease, obstructive sleep apnea, and obesity. Medications reported include amiloride, bisoprolol, finasteride, and zafirlukast. Relevant surgical history includes bilateral iliac stent placement in 2019.

On physical exam, it was noted that the patient was short of breath and had an increased work of breathing upon arrival into the room. Lungs were clear to auscultation bilaterally with normal chest wall expansion. The patient had a regular heart rate and normal rhythm. The patient’s lab work was notable for elevated brain natriuretic peptide (BNP, 2,013 pg/mL), creatinine (Cr, 1.66), and estimated glomerular filtration rate (eGFR, 41). Troponin was within normal limits.

**Figure 1:**
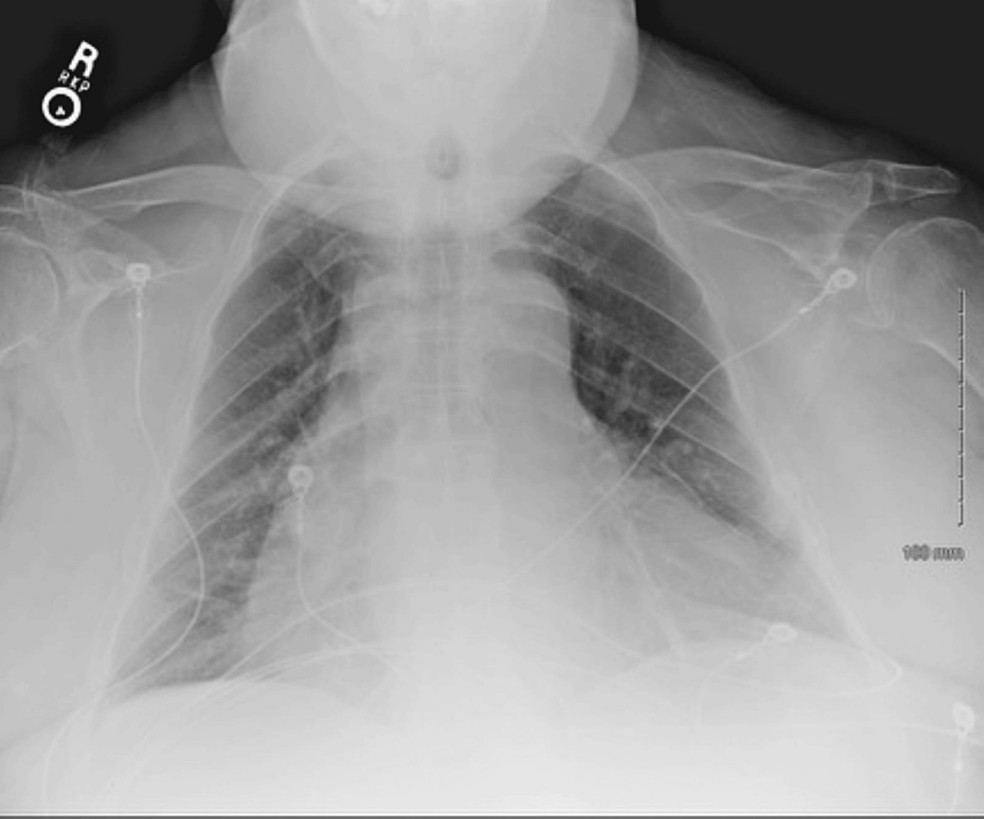
Chest radiograph AP view showing no acute cardiopulmonary findings associated with cardiomegaly. AP, Anterior-posterior

A chest radiograph was then performed in the ED and showed "no acute cardiopulmonary findings with cardiomegaly." The chest radiograph is displayed in Figure 1.

At this point, cardiology and cardiovascular surgery were consulted, and the patient was admitted to the cardiac intensive care unit (ICU) for further evaluation.

A right peripheral inserted central catheter (PICC) line was placed in the cardiac ICU. The patient was given fluids and put on a heparin drip. A transesophageal echocardiogram was performed and displayed a large iliac stent (reported 16x18 mm) lodged in the right ventricle from the level of the tricuspid annulus to the right ventricle apex and appears to be stuck in trabeculations. It is non-mobile and associated with severe tricuspid regurgitation as described. There is very severe tricuspid regurgitation due to the large iliac stent, preventing the tricuspid leaflet coaptation and flow directly through the stent regurgitating into the right atrium. The patient's TEE can be seen in Videos 1, 2. Video 1 shows the severe tricuspid regurgitation that the patient was experiencing. Video 2 shows the migrated iliac stent within the trabeculations of the right ventricle of the heart.

**Video 1:** 2D TEE color Doppler utilizing the mid-esophageal long axis view at 133 degrees showing severe tricuspid regurgitation through the right ventricle into the right atrium due to the large stent preventing tricuspid leaflet coaptation and flow directly through the stent regurgitating into the right atrium. 2D TEE, Two-dimensional transesophageal echocardiogram

**Video 2:** 2D TEE as shown from the mid-esophageal position long axis view at 120 degrees showing that there is an iliac stent (16x 18 mm) lodged in the right ventricle from the level of the tricuspid annulus to the right ventricular apex and appears to be stuck within the trabeculations. The stent appears to be non-mobile in the right ventricle. 2D TEE, Two-dimensional transesophageal echocardiogram

Due to the location of the stent and implantation within the trabeculations in the right ventricle, it was determined that this patient would need to have open-heart surgery to retrieve it. Snare retrieval was not an option because there was concern that portions of the heart may be susceptible to irreversible damage upon retrieval.

The patient had a complicated hospital stay. The patient was in the cardiac ICU for over a month. Within the span of the first two weeks, the patient’s creatinine climbed to the high 3’s, hemoglobin and hematocrit dropped, and the patient had a period of intubation. Multiple chest radiographs were performed during this patient's hospital stay. All of which were similar to that of Figure 1. Chest radiographs showed proper PICC line placement and were used to rule out other lung pathologies. All other chest radiographs mentioned were within normal limits and showed no changes. An esophagogastroduodenoscopy was performed emergently in the ICU, which was noted to show two gastric bleeding ulcers. No pictures were obtained due to the emergence of this diagnostic test. A coronary angiogram was performed and showed severe peripheral artery disease: 90-95% stenosis in the left anterior descending artery, 90-95% stenosis at the origin of the ramus, and large dominant and proximal 20% stenosis and distal 90-95% stenosis of the right circumflex artery. Videos and imaging of the coronary angiogram were not obtained. The left ventricle angiogram was not performed due to CKD-3b.

During the hospital course, the patient experienced a febrile episode with altered mental status. He experienced nausea and vomiting for which he was placed on ceftriaxone and tube feeds. He experienced acute cardiogenic shock, severe tricuspid regurgitation due to the migrated iliac stent, acute congestive heart failure with preserved ejection fraction, multivessel coronary artery disease, and lactic acidosis for which he was put on neo-synephrine for mean arterial pressure (MAP) support and dobutamine for ionotropic support. He was put on furosemide for increased edema. He suffered from a gastrointestinal bleed, acute blood loss anemia, gastroesophageal reflux disease, and dysphagia for which he was put on intravenous (IV) pantoprazole. The patient suffered from acute hypoxic respiratory failure for which he used supplemental oxygen and bilevel positive airway pressure (BiPAP). The patient was in acute renal failure due to decreased renal perfusion thought to be from a valvular defect known as severe cardiorenal syndrome. Additionally, the patient developed ICU myopathy and insomnia for which he was prescribed quetiapine.

The patient was in multiorgan failure. The cardiothoracic surgeon determined it would be far too dangerous to perform a six- to eight-hour surgery on this patient. The risk of surgery and snare removal outweighed the benefits. It was determined by all of the patient's following physicians that the patient would have a high likelihood of mortality if either intervention were to be performed.

During his time in the cardiac ICU, the patient was extensively followed by cardiology, gastroenterology, nephrology, the critical care team, and the hospitalist. He was carefully monitored hourly. Eventually, MAP support, dobutamine, IV pantoprazole, and ceftriaxone were discontinued. The patient’s creatinine came back to his baseline. He was discharged on tube feeds with multiple new medications, in addition to the reported history, to a skilled nursing facility in hopes of building enough strength for open-heart surgery. New medications were amiodarone, aspirin, insulin, levetiracetam, pantoprazole, quetiapine, montelukast, ipratropium, and Dulera.

While obtaining consent for this case report, after the patient’s hospital stay, the patient’s wife reported that the bilateral iliac stents were placed in 2019. She stated he had them placed due to “circulation issues” and could not exactly give an explanation as to why he had them placed. She reported that the patient had no history of DVTs. His wife also stated that he had not followed up with the proceduralist who placed his stents since 2019. In the skilled nursing facility, he had walked over 200 feet that day the follow-up call was made.

## Discussion

Our case describes a patient with “circulation issues” as known as venous insufficiency who presented with dyspnea, dizziness, and increased fluid retention. This patient experienced stent migration five years after stent placement to the right ventricle of the heart. Although rare, stent migration must be recognized because of its extremely serious, life-threatening complications. A patient with stent migration will typically present with nonspecific symptoms, such as dyspnea, heart palpitations, and chest pain [10]. They should be worked up promptly. All patients with previous stent placement should be aware of such symptoms and monitored closely. The stability and comorbidities of the patient are important to consider when discussing treatment options for stent retrieval because not all patients can withstand open-heart surgery and/or endovascular snare retrieval. Treatment options for this patient and most patients who experience stent migration include open-heart surgery and endovascular snare retrieval. Endovascular stent snare retrieval is preferred over open-heart surgery because it is less invasive. However, the treatment offered to each patient is individualized and depends on the location of the stent within the heart. Due to the patient's complicated hospital stay, it was determined that the patient would not be able to withstand a six- to eight-hour open-heart surgery. Due to the location of the migrated stent, lodged deep within the trabeculations of the right ventricle of the heart, it was determined that snare retrieval was also not an option. The patient's heart would be at risk for irreversible damage. The risks of this patient's treatment options outweighed the benefits. Neither treatment option was able to be performed.

Stent migration may occur because of selecting stents with inadequate length or diameter with most migrating stents being too short or too thin [11,12]. Additionally, it is thought that changes in the diameter of the veins during respiration, combined with the natural tendency of the stent’s expansion, can cause a notable decrease in the stent’s length. This occurs as the stent tends to expand more when the vessel is dilated and hence by the progressive increase in the diameter shortening [8]. A literature review conducted by Sayed et al. in 2022 from 1994 to 2020 identified 31 studies providing 54 events of venous stent migration with some data provided of the stent used in 47 of the stents used in the events. The most reported cases (82.6%) of migrating stents were ≤ 60 mm in length, 93.6% were ≤ 14 mm in diameter, and 41.6% occurred ≤ 30 days from stent placement, with only three reports of stents > 14 mm in diameter (3.6%) [11]. Additionally, Black et al. mentioned that, from the 2020-2023 FDA’s Manufacturer and User Facility Device Experience (MAUDE) database, a total of 67 stent migration cases have been reported from March 2019 to 2023, with more patients classified with May-Thurner or non-thrombotic iliac vein lesion (NVIL). More than half of the cases reported were stents 60-90 mm in length and ≤ 16 mm in diameter. Of the cases reported, the migrated stents were > 14 mm in diameter into the subclavian vein and inferior vena cava - less than half of the migrated stents reported occurred within 30 days of the procedure [12]. With that said, our patient was unique in that his stent was greater than 14 mm, 16x18 mm in diameter, and larger than most of the previous migrated stents, and his migratory event occurred long after stent placement (five years). The length of our patient’s stent was not reported. Potential ways to prevent stent migration is the proper usage of the gold standard modality, intravascular ultrasound (IVUS), to measure the vein’s area and diameter and/or to potentially increase the stent size so that the body does not uproot it.

Although we are uncertain of whether this patient’s proceduralist obtained the proper measurements due to the lack of history reported, it is important to mention that IVUS is a method that should be utilized to ensure the correct measurements of vein diameter and area of the target [3,13]. IVUS is the gold standard for confirmation and diagnosis of femoroiliocaval obstruction with a sensitivity of 90% [3,14,15]. It measures the luminal area of the vessel, with the most optimal stent sizes in the common iliac, external iliac, and common femoral veins being 16, 14, and 12 mm in diameter [14,15]. Other methods of diagnosis and vein measurement include duplex ultrasound (DUS) and computed tomography venography (CTV). DUS is noninvasive and readily available for the diagnosis of iliofemoral venous obstruction; however, it has some limitations. The limitations include the lack of consistent visualization of the iliac segments on account of bowel gas and or truncal obesity [14]. CTV was another option previously used to measure the size of the vein and percent stenosis; however, it was determined that CTV should be used in addition to IVUS because of limited diagnostic performance in identifying iliofemoral vein stenosis [15-17]. A study by Toh et al., using planimetric area computations of both CTV and IVUS, found a correlation coefficient of 0.57 (P < 0.005), with area measurements on CTV being larger than those obtained from IVUS [15,17]. A similar result was obtained in the study by Shammas et al. in 96 patients, where the authors noted that mean percentage stenosis on CTV and mean percentage stenosis on IVUS were not statistically different, despite an overestimation of minimal luminal area by CTV when compared with IVUS [15,16]. Proper methodologies and tools for vessel measurement may help avoid stent migration.

Additionally, close follow-up with the patient's proceduralist could have avoided such life-threatening complications. The patient’s wife stated that the patient failed to follow up post procedure. We are uncertain if stringent follow-up was relayed from the proceduralist to the patient. That said, endovascular follow-up guidelines are still lacking. According to the Society for Vascular Surgery Practice, there are no detailed or comprehensive guidelines implemented in the society after an open surgical and endovascular procedure that specify the most optimal approaches to testing methods, indications for reintervention, and follow-up intervals [18]. Perhaps more stringent recommendations would benefit both the patient and the physician. Close follow-up following one-year post operatively, with the use of sophisticated imaging methods such as CT, magnetic resonance (MR) angiography, and DUS, needs to be utilized more frequently to monitor the location of the stent [18]. If there were more stringent guidelines regarding endovascular stenting, and the patient was more aware of the importance of follow-up, he could have avoided a complicated hospital stay and the risks of life-threatening complications.

## Conclusions

Iliofemoral venous obstruction is a significant medical condition that can lead to severe and chronic symptoms, particularly when conservative management fails. The use of venous stenting has proven to be a reliable and effective treatment option for patients with chronic iliofemoral venous obstruction. Despite the overall safety and efficacy of stent placement, rare but serious complications such as stent migration can occur, leading to life-threatening conditions. In this case, we presented an 81-year-old male with a history of bilateral iliac stent placement who experienced stent migration to the right ventricle five years post procedure. This case underscores the critical nature of recognizing and addressing stent migration due to its potential for causing severe complications, including tricuspid regurgitation and right heart failure. Despite thorough evaluation and consideration of treatment options, the patient's complex medical condition and the risk associated with surgical and snare retrieval interventions precluded these procedures. This highlights the importance of patient-specific considerations and the need for careful planning and follow-up in managing venous stenting procedures.

To mitigate the risk of stent migration, accurate measurement of venous dimensions using IVUS during stent placement is essential. Furthermore, stringent follow-up protocols and patient education regarding the importance of post-procedural monitoring are crucial in preventing such complications. Our patient’s case illustrates the necessity for improved guidelines and consistent follow-up practices to ensure the long-term safety and efficacy of venous stenting. While venous stenting remains a cornerstone therapy for iliofemoral venous obstruction, vigilance in patient follow-up and adherence to best practices in stent selection and placement are vital. This case emphasizes the need for continuous improvement in procedural techniques and post-procedural care to prevent and manage complications effectively, ensuring better patient outcomes.
